# Hot mitochondria?

**DOI:** 10.1371/journal.pbio.2005113

**Published:** 2018-01-25

**Authors:** Nick Lane

**Affiliations:** Department of Genetics, Evolution and Environment, University College London, London, United Kingdom

## Abstract

Mitochondria generate most of the heat in endotherms. Given some impedance of heat transfer across protein-rich bioenergetic membranes, mitochondria must operate at a higher temperature than body temperature in mammals and birds. But exactly how much hotter has been controversial, with physical calculations suggesting that maximal heat gradients across cells could not be greater than 10^−5^ K. Using the thermosensitive mitochondrial-targeted fluorescent dye Mito Thermo Yellow (MTY), Chrétien and colleagues suggest that mitochondria are optimised to nearly 50 °C, 10 °C hotter than body temperature. This extreme value questions what temperature really means in confined far-from-equilibrium systems but encourages a reconsideration of thermal biology.

Self-respecting scientists might be inclined to back away from the claims made by Chrétien and colleagues in *PLOS Biology* this week: mitochondria in endothermic mammals operate optimally at close to 50 °C [1]. That is a radical claim, and if it is true, how come we didn’t know something so important long ago? If it isn’t true, then why is it figuring so prominently in the pages of a respected journal and how did it get through peer review? The answer is that this finding challenges many of our cherished beliefs, whether it is right or wrong, or perhaps more probably somewhere in between.

## Molecular probes

The study uses the thermosensitive dye Mito Thermo Yellow (MTY), which accumulates in the mitochondrial matrix depending on the membrane potential [2]. Within the matrix, MTY appears to bind to the matrix isoform of aldehyde dehydrogenase 2 (ALDH2), although its exact submitochondrial location is uncertain [3]. The fluorescence of MTY falls by around 2.7% for each 1 °C rise in temperature in aqueous solution; hence, in principle, this allows calibration of the temperature right next to the main source of heat in cells, the mitochondrial inner membrane [1,2].

Many other variables could potentially affect its fluorescence, notably pH, oxygen tension, superoxide production, and membrane potential, but Chrétien and colleagues controlled for these variables as well as can readily be done given that MTY quickly leaks out of uncoupled mitochondria, abolishing a meaningful signal [1]. That precluded a closer analysis of other possible confounding factors, such as changes in Ca^2+^ concentration. Like other rosamine-based dyes, MTY fluorescence can be quenched by aggregation at high concentrations, but the low nM concentrations used in the study should have precluded quenching. The major cause for concern from earlier work is that the change in fluorescence can be sensitive to the tissue type; the fall in fluorescence in brown adipocytes was 2.0% per 1 °C rise in temperature [2] for reasons that presumably reflect the dye’s response to a specific environment.

## Mind the gap

In the human embryonic kidney cells 293 (HEK293) and primary skin fibroblast cells studied here [1], the response of MTY to respiration seems intuitively reasonable: actively respiring mitochondria heat up, various respiratory inhibitors cool them down—as does elimination of most mitochondrial DNA (mtDNA)—and expression of an engineered alternative oxidase overcomes inhibition by cyanide, warming them up again.

All this makes good sense, except for the fact that the recorded mitochondrial temperatures were some 10 °C above the surrounding water bath, which was maintained at 38 °C [1]. This difference is extreme and seems barely credible, especially given robust critiques [4,5] of earlier work using different (nonmitochondrial) thermosensitive probes that purported to show smaller (several K) subcellular heterogeneities in temperature [6,7]. The criticisms were based on apparent physical constraints such as calculated rates of heat transfer through aqueous media, which suggest that gradients of more than 10^−5^ K could not be sustained across a cell [4,5]. This has become known as the 10^5^ gap [8]—the difference between predictions grounded in physics and apparent empirical measurements. Chrétien and colleagues widen the gap to 10^6^.

**Fig 1:**
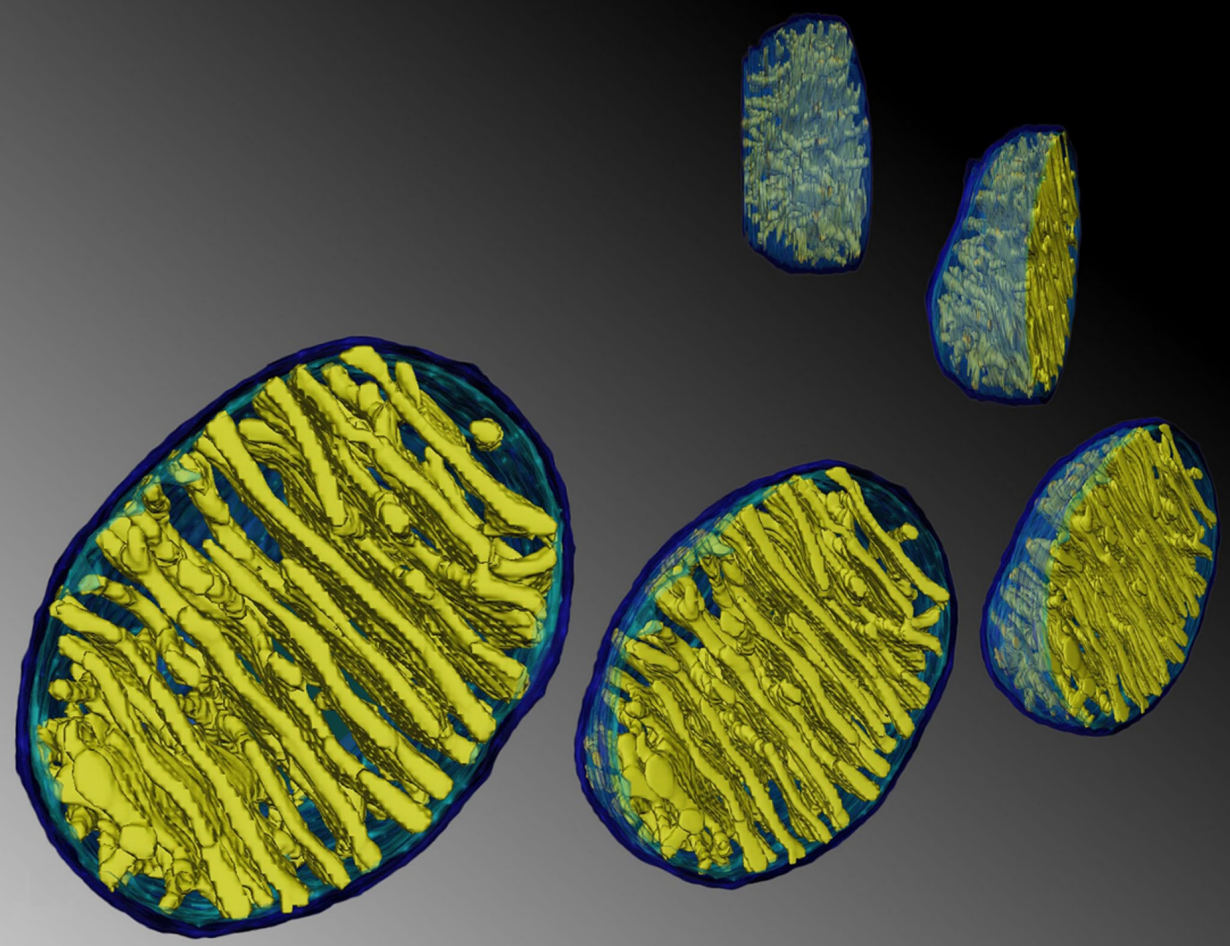
Mitochondria as radiators: Parallel arrays of closely appositioned cristae membranes could heat the mitochondrial matrix. (Image courtesy of Terrence G. Frey, San Diego State University, used in doi: 10.1371/journal.pone.0004604).

But are these calculations correct? There are reasons to question some of the parameters used. For simplicity, most physical calculations assume that mitochondria are membrane-bound spheres, with heat production occurring across the surface of the spheres [4,5]. But that is far from the truth; in light of Chrétien and colleagues’ findings [1], the mitochondria shown in Fig 1 look a lot like radiators. Heat production occurs across the cristae membranes, which typically lie in parallel, potentially retaining heat within the matrix.

Mitochondria are often fused into large branching networks that probably retain heat better than smaller individual compartments. That is because the mitochondrial inner membrane is not a normal lipid bilayer but is densely packed with proteins, virtually glued together by unusual lipids such as cardiolipin. It normally maintains an electrochemical membrane potential of up to −200 mV. I am not aware of measurements of heat conductance across the inner membrane, but it could differ substantially from the values used for water or ‘normal’ lipid bilayers. In contrast, water and heat might pass through the mitochondrial outer membrane almost without obstruction.

## Cell power

The values of cell power used—typically 100 pW—are also not representative. Based on measured metabolic rates of protists, a mean value of 2,000 pW is closer to the truth [9], whereas large amoebae, such as *Amoeba dubia*, have a metabolic power of up to 10,000 pW [9]—which alone closes the gap by two orders of magnitude.

There are large differences in heat production between idling mitochondria, which are presumably close to thermal equilibrium, and maximal respiratory rates (as measured by Chrétien and colleagues). A beautiful example is the heat produced by large insects such as locusts when flying, which warms their thorax by 5 °C to 15 °C within minutes, regardless of the ambient temperature [10]. Locusts are obliged to stop flying when the temperature of the thorax surpasses about 40 °C, which does not take them long [10]. This substantial mitochondrial heat production sustains temperature gradients of nearly 1 °C per mm, corresponding to about 0.05 °C across individual cells, as measured using old-fashioned thermocouples [10]. These high temperatures are limited to the thorax; the temperature of the abdomen barely changes during flight [10]. Even steeper heat gradients across cells must exist in between.

So, fast respiration easily pushes animals out of equilibrium in terms of their rate of heat loss. The limits on heat production are imposed not by mitochondrial activity but by behavioural changes such as quitting flying, which implies that heat loss is much slower than the maximal rates of heat generation.

The fact that Chrétien and colleagues found that the temperature optima for several mitochondrial enzymes was also close to 50 °C reinforces that view, although it is well known that temperature doubles the metabolic rate for every 10 °C rise in temperature until the enzymes begin to denature [11]. Whether the mitochondrial enzymes are genuinely optimised for close to 50 °C or just operate faster at that temperature before they fall to pieces is not clear. The cellular context—chaperones, ion concentration, and so on—could make a big difference to protein stability.

## The meaning of temperature

Is it feasible, then, that the changes in temperature really are as great as those measured by Chrétien and colleagues? That seems doubtful too and begs another question: what does temperature mean in the confined spaces of the mitochondrial matrix? Temperature is a statistical equilibrium property of a large population of molecules—it is a measure of the average kinetic energy, or molecular motion, and by definition there can’t be very steep heat gradients, or temperature could not be measured. An equivalent question relates to pH and is perhaps easier to visualise. If a single proton binds to a water molecule in a confined membrane channel, what is the pH? The question has no meaning because pH is an emergent property of proton concentration in bulk solution.

In the case of temperature, the mitochondrial matrix is densely packed with proteins. Their conformational states are stabilised by structural water, which by definition is less free to move but must move when a protein changes its conformation. How much water is free to move, and to what extent is the vibrational excitement of fixed water molecules passed on to the molecular motion of free water in the matrix? Earlier work shows that viscosity alone can affect MTY fluorescence, even in the much simpler system of polyethylene glycol in water [2], which doesn’t begin to capture the complexity of the mitochondrial matrix.

## Molecular machines

What about turbulent motion in the vicinity of proteins? Respiratory proteins are extraordinary nanoscale molecular machines that can change their conformational state hundreds of times per second; the ATP synthase rotates at up to 400 times per second [12].

There is an interesting trend across membrane-integral respiratory proteins in metazoans whereby many of the hydrophobic amino acids in animals with low metabolic rates such as nematodes are replaced with serine and threonine residues in more active animals, notably birds and mammals [13]. Serine and threonine residues can form extensive networks of weak hydrogen bonds between transmembrane helices, which could facilitate rapid switches between the conformational states involved in proton pumping [14]. They might also help stabilise the assembly of supercomplexes [14], which Chrétien and colleagues show are more robust at higher temperatures than individual respiratory complexes [1].

## Turbulence and heat

All these swiftly changing conformational states in tens of thousands of molecular machines per mitochondrion might be detected as a higher temperature by MTY. Its sensitivity to temperature depends in part on the rotational freedom of its substituents [2]. The strength of the fluorescence signal is affected by collisional quenching and the fast, turbulent motion of neighbouring molecules, but neither of these necessarily always or only relates to temperature. Could it be that molecular machines like the rotating ATP synthase increase the turbulent motion of molecules in their vicinity, which MTY might register as heat? Conversely, could the lower sensitivity of MTY in brown adipocytes [2] relate to mitochondrial uncoupling, whereby the inactivity of the ATP synthase produces less turbulent motion? While brown adipocyte mitochondria generate heat through uncoupling, which increases collisional quenching and turbulence, uncoupling could dampen the contribution of molecular machines to overall turbulence, altering the apparent sensitivity of MTY to temperature.

We need to know a lot more about both the specific behaviour of MTY and its exact location within the mitochondrial matrix before we can come to any firm conclusions about ‘temperature’. In the meantime, I doubt that the 10 °C temperature difference should be taken literally.

But it should be taken seriously. Heat has fallen out of fashion in biology. The origin of endothermy was once a turbulent subject [15], but it scarcely raised a bubble even in the furore about allometric scaling of metabolic rate with body size [16]. Endothermy is generated by the simple expedient of having about five times as many mitochondria in the visceral organs as equivalent poikilotherms [17], which, in evolutionary terms, may have originally been associated with greater stamina and a higher field metabolic rate [17,18]. The dichotomy between male and female development might relate to differences in metabolic rate—the ‘hot male’ hypothesis [19]. Remarkably, the faster growth of embryonic male mammals is the earliest detectable difference between the sexes, even before the SRY gene is expressed [19]. Small differences in heat generation by mitochondria might influence the risk of diseases ranging from diabetes and male infertility [20] to cancer [21] and the rate of ageing [22].

Whether or not all these ideas are correct, the distribution and heat generation of mitochondria within cells should be taken much more seriously. Chrétien and colleagues bring this important subject back to centre stage, which is exactly where it should be.

## Abbreviations

ALDH2: aldehyde dehydrogenase 2
HEK293: human embryonic kidney cells 293
mtDNA: mitochondrial DNA
MTY: Mito Thermo Yellow
